# The main cause of tibial prosthesis malalignment after total knee arthroplasty in Southern Chinese population

**DOI:** 10.1016/j.heliyon.2024.e25447

**Published:** 2024-01-29

**Authors:** Lili He, Congcong Wu, Junzhe Lang, Lei Chen, Peng Wu

**Affiliations:** The First Affiliated Hospital of Wenzhou Medical University, Nanbaixiang street, Ouhai district, Wenzhou city, Zhejiang province, 325200, China

**Keywords:** Total knee arthroplasty, Malalignment, Tibial bowing, Medial proximal tibial angle, Tibial plateau shift angle

## Abstract

**Objectives:**

This study aimed to determine the occurrence rate of malalignment of tibial prosthesis and explore the influencing factors.

**Methods:**

296 patients from Southern China who underwent total knee arthroplasty (TKA) were selected as the research objects. Their general demographic data were recorded. The tibial bowing angle (TBA), tibial length, medial proximal tibial angle (MPTA), tibial plateau shift angle (TPSA), tibial bone loss, lateral distal tibial angle, and overall width of tibial plateau and widths of medial and lateral tibial plateau were measured before TKA. The tibial component coronal alignment angle (TCCA) was measured after the operation. Malalignment of the tibial prosthesis was defined as TCCA <87° or TCCA >93°. Tibial bowing was indicated by TBA >2°, and lateral bowing was recorded as +. The correlations of TCCA with demographic data and pre-operation imaging measurement parameters were statistically analyzed.

**Results:**

Bivariate correlation analysis revealed negative correlations between TCCA and TBA (r = −0.602, P < 0.001) and TPSA (r = −0.304, P < 0.001), and a positive correlation with MPTA (r = −0.318, P < 0.001). Multivariate linear regression analysis demonstrated a significant negative correlation between TCCA and TBA (P < 0.001). The occurrence rate of malalignment of tibial prosthesis was 12.37 %. The occurrence rates of malalignment were 22.54 % in the tibial bowing group and 6.87 % in the non-tibial bowing group, showing statistical differences (P < 0.001).

**Conclusion:**

The malalignment rate of tibial prosthesis among Southern Chinese patients is relatively high, possibly attributed to the tibial anatomy anomalies, particularly the tibial bowing. The entry point should be determined based on tibial morphology.

## 1 Introduction

Precise alignment of lower limbs is imperative for optimal function following total knee arthroplasty (TKA) [1]. Any extroversion or introversion of prosthesis relative to normal mechanical axes for more than 3° is defined as malalignment [2] (outlier) according to the principles of mechanical alignment technique, which results in imbalanced loads between the internal and external facets of the prosthesis and accelerated wearing of the prosthesis [3,4]. Consequently, the longevity of the prosthesis is adversely affected.

Most knee prostheses available in the market are designed on the basis of imaging data from Caucasian population. Asians have differences with Caucasians in terms of the anatomy of femur and tibia [[5], [6], [7], [8], [9], [10]]. These anatomical variations may impact the precision of prosthetic implantation, potentially leading to malalignment of the prosthesis. At present, many studies on femur have been conducted [5,8,11,12]. However, few studies have focused on the demographic or radiographic parameters of tibial prosthesis implantation on the coronal plane. Yau et al. [5] found that tibial coronal bowing caused malalignment of tibial prosthesis, but they solely implemented an imaging simulation study utilizing intramedullary localization technique. Kobayashi et al. [8] found problems of tibial bowing, but they did not further discuss the influences of tibial bowing on the tibial prosthetic alignment. Chang et al. [12] and Nagamine et al. [6] revealed anomalies in tibial anatomy among patients with osteoarthritis and emphasized the importance of attention to these anomalies during TKA. However, they did not provide a comprehensive discussion on the specific influences of tibial anatomy anomalies on the prosthetic alignment.

To date, there is an absence of comprehensive studies concerning the malalignment rate of tibial prosthesis and the causing factors in Chinese patients with osteoarthritis. We hypothesized that these tibial anatomical abnormalities may contribute to postoperative malalignment of tibial prosthesis during primary TKAs, particularly when extramedullary localization points of the tibia were positioned at the center of anterior and posterior intercondylar eminence. Therefore, the aims of this retrospective study were (1) to investigate the occurrence rates of malalignment of tibial prothesis in southern Chinese patients with knee osteoarthritis; (2) to identify the tibial abnormities among southern Chinese patients; (3) to assess the impact of tibial morphology on malalignment of tibial prosthesis.

## 2 Materials and methods

### 2.1 Patients

A total of 296 patients who underwent TKA at the orthopedics department between January 1, 2020, and June 1, 2021 were reviewed. The inclusion criteria are as follows: (1) knee osteoarthritis, invalid in conservative treatment; (2) undergoing TKA for the first time. The exclusion criteria are as follows: (1) rheumatoid arthritis, traumatic arthritis, and gouty arthritis; (2) a history of femur or tibial corrective osteotomy; (3) full-length films taken in non-rotating neutral position, which could compromise image measurement; (4) a history of severe traumas to knee joint; (5) serious flexion contracture distortion >20° before operation, leading to unacceptable measurement errors [13]; and (6) valgus knees deformity [14,15]. In the present study, a total of 202 patients (202 knees) were included. The age, gender, height, surgical side, and body weight of patients were recorded, and BMI was calculated. This study was approved by the ethics committee of Wenzhou Medical University first affiliated hospital (Ethics Approval No.: 2021R070).

### 2.2 Surgical technique

All operations were performed by two senior surgeons and adhered to the principles of mechanical alignment technique, which stipulates hip-knee-ankle axe (HKA), femoral and tibial prostheses should be within 3° relative to mechanical axe. The extramedullary localization method was employed during tibial osteotomy. The extramedullary localization points of the tibia were positioned at the center of anterior and posterior intercondylar eminence. With references to the line of force at lower limbs centered at the anterior crest of the tibia and ankle point center, the rod of line of force was adjusted, and then the osteotomy plate was fixed. Once optimal results were achieved, the prostheses were implanted and compacted. A full-length film of knee joint at the standing position was taken before the discharge from day 3 to 1 week post-operation. The prostheses utilized in the patients were either Zimmer NexGen LPS (Zimmer, Warsaw, USA) or Stryker NRG (stryker, Kalamazoo, State of Michigan, USA). The selection was based on comprehensive considerations such as surgical intent, cost, and the specific prosthetic requirements of individual patients during the operation.

### 2.3 Radiologic assessment

**Fig. 1:**
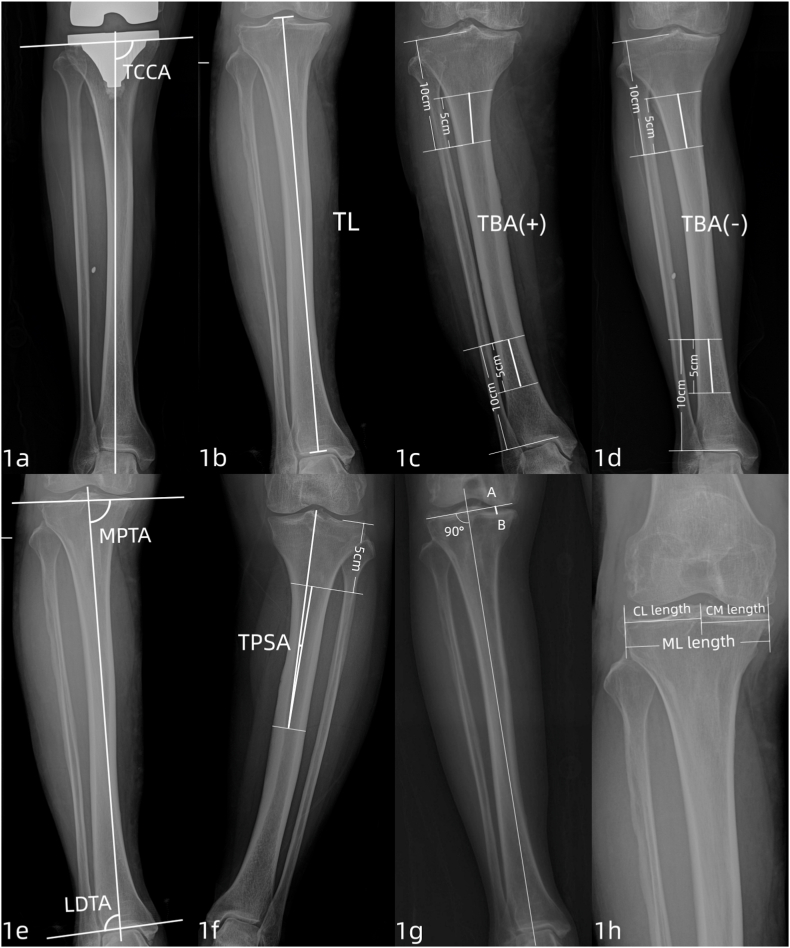
Imaging measurement is performed on a full-length hip-to-ankle radiograph. **1a** TCCA, tibial component coronal alignment anglel; **1b** Tibial length; **1c** TBA, tibial bowing angle, the figure shows lateral bowing; **1d** TBA, tibial bowing angle, the figure shows medial bowing; **1e** MPTA, medial proximal tibial angle; LDTA, lateral distal tibial angle; **1f** TPSA, tibial plateau shift angle; **1g** tibial boss loss; **1h** ML length, medial to lateral length; MC length, medial to center length; CL length, center to lateral length.

Post-operation tibial component coronal alignment angle (TCCA) [16] was measured by the same author using Image-Pro Plus software on the Picture Archiving and Communication System (PACS). TCCA is defined as the internal angle between the mechanical axis of tibia and the lower surface tangent of the tibial prosthesis (see Fig. 1a). Malalignment of tibial prosthesis is defined as TCCA >93° or TCCA <87°.

On the pre-operation full-length film taken in the standing position, the same author conducted measurements on the following indices. (1) Tibial length [12] is the distance from midpoint of tibial intercondylar eminence to distal joint surface of tibia (see Fig. 1b). (2) Tibial bowing angle (TBA) [12] is the included angles between the connected line from tibial midpoint at 5 cm to that at 10 cm below the external plateau and the connected line from tibial midpoint at 5 cm to that 10 cm above the distal tibial joint (see Fig. 1c and d). Lateral bowing of tibia was denoted as “+” while medial bowing was denoted as “-”. TBA < −2° or >2° is considered as obvious tibial bowing [5]. (3) Tibial plateau shift angle (MPTA) [17] refers to the internal included angle between the tangent line of the tibial plateau and the mechanical axis of tibia (see Fig. 1e). (4) Tibial plateau shift angle (TPSA) [12] is the included angle between the connected line from the midpoint of the middle section of tibia to the plateau midpoint and the connected line from the midpoint of the middle section of tibia to the midpoint of tibia 5 cm below the lateral plateau (see Fig. 1f). Inward shift of midpoint of tibial plateau relative to the tibial anatomy was denoted as “+”. (5) Tibial bone loss [11] refers to the distance from the tangent line of the highest point of the lateral platform perpendicular to the mechanical axis of tibia to the lowest point of the medial plateau (see Fig. 1g). (6) Lateral distal tibial angle (LDTA) [18] denotes the lateral angle formed by the tibial mechanical axis and the tangent line of the distal tibial joint surface (see Fig. 1e). (7) In the overall width of tibial plateau and widths of medial and lateral sides of the plateau [19], the tibial plateau width is the distance from the edges of lateral plateau, excluding osteophyte, to the edges of medial plateau. The medial plateau width is the distance from the midpoint of tibial intercondylar spine to the edges of medial platform. The lateral plateau width is the distance from the midpoint of tibial intercondylar spine to the edges of lateral platform (see Fig. 1h). The results are presented in Table 1.

**Table 1:** Preoperative and postoperative radiologic parameters.

	Means	SD	Min	Max
Preoperative				
TBA (°)	0.32	2.43	−5.94	7.45
Tibial length (cm)	33.67	2.41	28.60	40.67
MPTA(°)	84.98	2.82	77.87	93.09
TPSA(°)	1.00	1.22	−2.33	3.89
Tibial bone loss (mm)	7.25	3.57	0.5	18.62
LDTA (°)	91.25	2.87	85.35	100.35
ML length (mm)	73.73	5.21	60.13	90.35
MC length (mm)	36.26	2.77	29.98	43.84
CL length (mm)	37.47	4.30	20.69	47.45
Postoperative				
TCCA(°)	90.20	1.22	85.09	94.45

TBA tibial bowing angle, MPTA medial proximal tibial angle, TPSA tibial plateau shift angle, LDTA lateral distal tibial angle, ML medial to lateral width, MC medial to center, CL center to lateral, TCCA tibial component coronal alignment angle.

### 2.4 Statistical analysis

Counts were expressed by means and standard deviations. The overall outlier rate was statistically summarized. A bivariate correlation analysis was conducted to examine the relationship between tibial component coronal alignment angle (TCCA) and demographic data as well as radiographic parameters. Pearson's correlation analysis was applied for continuous measurement data, while Spearman's correlation analysis was employed for categorical variables (Table 2). Statistically significant indices, which were represented by bi-variate correlation analysis and may cause malalignment of tibial prosthesis, were subjected to the multivariate linear regression analysis. Significant indices were screened by stepwise regression method (Table 3). P < 0.05 indicated statistical significance. Data were statistically analyzed using IBM SPSS Statistics 19 statistical software.

**Table 2:** Correlation between tibial component coronal alignment angle (TCCA) and variables.

Parameters	R value	P value
Demographic data		
age(years)	0.006	0.932
Gender	−0.097	0.171
Operation side	0.005	0.947
Height (m)	−0.073	0.304
Weight (kg)	−0.135	0.055
BMI (kg/m2)	−0.105	0.136
Radiologic parameters		
Tibial length（cm）	0.006	0.937
TBA（°）	−0.602	<0.001*
MPTA(°)	0.318	<0.001*
TPSA(°)	−0.304	<0.001*
Tibial bone loss (mm)	−0.094	0.182
LDTA (°)	−0.004	0.950
ML length (mm)	−0.085	0.231
MC length (mm)	−0.086	0.222
CL length (mm)	−0.047	0.506

TBA tibial bowing angle, MPTA medial proximal tibial angle, TPSA tibial plateau shift angle, LDTA lateral distal tibial angle, ML medial to lateral width, MC medial to center, CL center to lateral. *P < 0.05 indicates statistically significant difference.

**Table 3:** Correlation between tibial component coronal alignment angle (TCCA) and variables（multivariate linear regression analysis）.

Parameters	t value	P value
TBA	−9.397	<0.001
MPTA	1.731	0.085
TPSA	−1.927	0.055

TBA tibial bowing angle, MPTA medial proximal tibial angle, TPSA tibial plateau shift angle. *P < 0.05 indicates statistically significant difference.

## 3 Results

Out of the 202 cases, 141 knees belonged to female patients, and 61 knees belonged to male patients. Surgeries were performed on the left side in 103 cases and on the right side in 99 cases. The mean age was 69.51 ± 7.16 years (SD). The average weight was 63.72 ± 9.76 kg (SD). The mean height was 1.59 ± 6.51 m (SD). The mean BMI was 25.11 ± 3.55 kg/m^2^ (SD). The mean values of preoperative and postoperative radiologic parameters are provided in Table 1.

Among the 202 cases of TKA, 25 exhibited malalignment of tibial prosthesis after operation (occurrence rate of 12.37 %), 15 demonstrated introversion of tibial prosthesis (TCCA <87°, 7.43 %), and 10 showed extroversion of tibial prosthesis (TCCA >93°, 4.95 %).

According to bi-variate correlation analysis, TCCA exhibited a robust correlation with TBA, displaying a correlation coefficient of −0.602 (P < 0.001). TCCA demonstrated a weak correlation with MPTA, indicated by a correlation coefficient of 0.318 (P < 0.001). Additionally, TCCA showed a weak correlation with TPSA, presenting a correlation coefficient of −0.304 (P < 0.001). However, TCCA demonstrated poor correlations with other variables (P > 0.05, Table 2).

According to multivariate linear regression analysis, tibial bowing emerged as the most significant factor contributing malalignment of tibial prosthesis after the operation (t = −9.397, P < 0.001, Table 3).

The proportion of tibial bowing (TBA < −2° or >2°) in the total studied cases was statistically analyzed to further investigate the impact of tibial bowing on the malalignment of tibial prosthesis. Additionally, all study cases were categorized into bowing group and non-bowing group based on the presence of obvious tibial bowing. The results revealed that 71 cases exhibited tibial bowing (35.15 %), including 44 cases of lateral bowing (21.78 %) and 27 cases of media bowing (13.37 %).

According to the statistical analysis of outlier rates, 16 (22.54 %) cases in the bowing group and 9 (6.87 %) in the non-bowing group had malalignment of prosthesis, respectively, indicating statistically significant differences (χ2 = 159.307, P < 0.05). Details are presented in Table 4.

**Table 4:** Comparison of tibial component malalignment between tibial bowing group and tibial non-bowing group.

Group	No.	Alignment	
		Non-outliers	Outliers
Bowing	71	55	16
Non-bowing	131	122	9
Value		χ2 = 159.307	
P		<0.001	

*P < 0.05 indicates statistically significant difference.

## 4 Discussions

This was a comprehensive study to investigate the malalignment rate of tibial prosthesis among southern Chinese patients and the causing factors. The results confirmed a relatively high malalignment rate, attributing it to tibial anatomy anomalies within the studied groups, particularly tibial bowing.

An essential objective of TKA is to restore the proper alignment of lower limbs. Although controversies still remain [10,20,21], the deviation of alignment of tibial prosthesis from the mechanical axis of tibia for more than 3° is defined as malalignment according to the principles of mechanical technique. The present study revealed an overall malalignment rate of 12.37 %, comprising 7.43 % of introversion and 4.95 % extroversion.

The relatively high malalignment of tibial prosthesis may be linked to the substantial prevalence of tibial anatomy anomalies in Asians. Yau et al. reported that 32 % of individuals in Hong Kong with osteoarthritis experienced tibial bowing. And imaging simulation studies indicated that tibial bowing could lead to malalignment of tibial prosthesis. Kim et al. [19] found that 14.3 % of Korean cases had tibial bowing. While Kobayashi et al. [8] recognized 21 cases of tibial bowing, including 11 lateral bending cases and 10 medial bowing cases, in 70 Japanese cases,. The occurrence rate of tibial bowing in the current study was 35.15 %, comprising 21.78 % lateral bowing and 13.37 % medial bowing, aligning with the findings of the aforementioned studies. Previous studies have highlighted that Asians with knee osteoarthritis tend to have smaller MPTA compared to Caucasians. In this study, the MPTA was 84.98°, consistent with reported values in Koreans (85°) [19], Japanese (83°) [6], and males (84.6°) and females (85.1°) from Hong Kong. Additionally, the tibial plateau shifted from the mechanical line of tibia to some extent [6,7,12]. The TPSA in the present study was approximately 1.0°, resembling the average TPSA (2.0°) reported by Nagamine et al. and the results in males (1.0°) and females (0.9°) reported by Chang et al. [12]. Finally, several factors, such as unequal widths of medial and lateral plateaus [6,19] and bone loss [11] may impact the line of force at the prosthesis. while some scholars have recognized the influences of tibial anatomical anomalies on the line of force at prosthesis [5,6,8,11,12,14,19], comprehensive associated studies are yet to be reported.

This study identified tibial bowing as the primary cause of malalignment of the tibial prosthesis. Lateral bowing of tibia tends to result in introversion implantation of the prosthesis, while medial bowing of tibia tends to lead to extroversion implantation of prosthesis. Follow-up analysis further demonstrated significant differences in the malalignment rate of tibial prosthesis between the bowing group and the non-bowing group. This finding may be attributed to two main points: (1) during localization, extramedullary localization method and proximal midpoint localization of the intercondylar spine were basically employed. The mechanical axis was referenced to the anterior crest of tibia and ankle point center. When tibial bowing was present, the anterior crest of the tibia did not align with the mechanical axis. Locating the ankle point center accurately is relatively challenging, surgeons often rely on the anterior crest of the tibia. When using the anterior crest of the tibia as the osteotomy reference for the mechanical axis, it results in suboptimal positioning of the prosthesis. (2) During osteotomy, Patients with lateral bowing typically require more osteotomy volume on the lateral tibial plateau compared to normal cases to compensate for the effects of tibial bowing. If surgeons are unaware of the presence of tibial bowing, they might intentionally reduce the osteotomy volume on the lateral tibial plateau, fearing excessive osteotomy on the lateral side. This cautious approach may lead to introversion of the tibial prosthesis. On the contrary, medial bowing of tibia leads to extroversion implantation of the prosthesis.

Moreover, the bi-variate correlation analysis revealed a positive correlation between MPTA and TCCA and a negative correlation between TPSA and TCCA. In other words, patients with large MPTA or TPSA may be more prone to introversion of tibial prosthesis. The underlying causes align with the second cause of osteotomy in the abovementioned: surgeons often intentionally reduce the osteotomy of external tibial plateau to prevent excessive osteotomy at the lateral side. Sung-Mok et al. [19] also recommended that if patients have small MPTA, the entry point should be adjusted lateral appropriately to avoid introversion. However, in the multivariate linear regression analysis, the influences of MPTA and TPSA on TCCA disappeared. Tibial bowing (from −5.94° to 7.45°) in patients with osteoarthritis may have weakened the effects of MPTA (from 77.87° to 93.09°) and TPSA (from −2.33° to 3.89°) (Table 1). Matsumoto et al. [7] discovered that early stage of knee osteoarthritis begins with medial shifting of tibial plateau (increase in TPSA) and then progresses to medial platform compression (increase in MPTA). In accordance with this study and previous studies, the development of osteoarthritis was deduced. Compression of tibial plateau (increase in MPTA) and tibial bowing began to manifest after the inward shifting of tibial plateau (increase in TPSA) reached a certain extent. Eventually, TBA emerged as the major factor and thereby mostly influenced the prosthetic alignment. TL, plateau bone loss, and width of plateau showed no correlations with TCCA, indicating that they are not the major contributors to the malalignment of the tibial prosthesis.

Given the relatively high prevalence of tibial bowing and small MPTA and large TPSA in Chinese patients, the recommendations are proposed to achieve corrective alignment of the tibial prosthesis: (1) full-length films of lower limbs should be obtained before the operation [5] to identify anatomical anomalies, such as tibial coronal bowing, small MPTA, and medial shifting of tibial plateau [12]. Chang et al. [12] also proved that short film could not replace full-length film in pre-operation evaluation of patients with osteoarthritis. (2) Given that anatomical anomalies are universal in patients with osteoarthritis, extramedullary osteotomy is generally suggested as a conventional treatment, while intramedullary osteotomy should be avoided [5]. (3) The proximal localization entry point of tibia should be determined based on pre-operation imaging parameters [6]. For patients with lateral bowing, small MPTA, or large TPSA, the proximal plateau localization entry point should be adjusted laterally. On the contrary, for patients with medial bowing, large MPTA, or small TPSA, the proximal plateau localization entry point should be adjusted medially [19]. (4) During operation, the detection technology reported by Ma et al. [22] could be employed, where the extramedullary rod is applied several times to test the alignment of prosthesis. If any bowing is present, the increased osteotomy volume or the use of shim could be employed to ensure proper alignment. (5)computer navigation could be applied upon the discovery of serious distortion in pre-operation radiographic examination [8,11], which could increase accuracy.

This study also has some limitations. First, it was a retrospective study conducted at a single medical center and involved only the southern Chinese population. Our results may not be applicable to other races. Second, the expertise of surgeons may influence the research results. All patients were operated on by the two senior surgeons to minimize errors as much as possible. Third, the full-length film may be subject to rotation and flexion deformity distortion. If the flexion deformity exceeds 20°, it may cause unacceptable measurement errors [13]. Therefore, patients with severe flexion contracture were excluded. Even when the rotation exceeded 40°, the relevant measurement error was only 2.5° [23]. Therefore, the measurement data on the standard full-length film at standing positions could provide convincing results. But future studies involving computed tomography would be more reliable than the study using simple radiographs. Fourthly, no single prosthesis was applied for studies. However, based on the standard operational process of total knee arthroplasty, the difference in prosthesis should not affect the results. Fifthly, the subjects in this study were knee arthroplasty patients using the mechanical alignment technique only, so the results cannot be extrapolated to other techniques, such as kinematic alignment technique. Sixthly, the sample size of this study is relatively small, which leads to a scarce number of patients with malalignment. This might affect the statistical power of the comparison between the bowing and non-bowing groups. Nevertherless, combined with regression analysis results, the conclusion that tibial bowing is the main cause of malalignment of tibial prosthesis is reliable. Lastly, as objective of this study was to explore the influencing factors of tibial prostheses, we didn't include the HKA and femoral deformation. The treatment of tibial is independent of that of femur, which wouldn't compromise the results.

## 5 Conclusion

A notable occurrence of tibial prosthesis malalignment was observed in southern Chinese patients with osteoarthritis. Such rate was the collaborative result of tibial coronal bowing cases, large MPTA, and medial shifting of tibial plateau. Among these factors, tibial bowing emerged as the primary cause. Given the prevalence of these tibial abnormalities in southern Chinese population with osteoarthritis, obtaining a full-length film in a standing position is imperative for preoperative planning. And the entry point should be determined based on tibial morphology. For patients with lateral bowing, small MPTA, or large TPSA, which are common characteristics, the entry point should be adjusted lateral appropriately. These findings provide valuable insights for surgeons undertaking tibial bone resection during primary TKAs.

## Ethical approval

This study was approved by the ethics committee of Wenzhou Medical University first affiliated hospital (Ethics Approval No.: 2021R070).

## Informed consent statement

Agreements for academic use of clinical and radiographic date were obtained from the patients by the hospital at the time of their hospitalization, and no identifiable information of the participants is included in the manuscript.

## Study design

Retrospective clinical study without control group.

## Level of evidence

Ⅳ，retrospective study without control group.

## Ethics approval and consent to participate

This study was reviewed and approved by the ethics committee of Wenzhou Medical University first affiliated hospital, with the approval number: 2021R070. Agreements for academic use of clinical and radiographic date were obtained from the patients by the hospital at the time of their hospitalization, and no identifiable information of the participants is included in the manuscript.

## Data availability statement

Data will be made available on request.

## CRediT authorship contribution statement

**Lili He:** Writing – review & editing, Writing – original draft, Formal analysis, Data curation, Conceptualization. **Congcong Wu:** Formal analysis, Data curation. **Junzhe Lang:** Formal analysis, Data curation. **Lei Chen:** Methodology. **Peng Wu:** Writing – review & editing, Writing – original draft, Supervision, Methodology, Conceptualization.

## Declaration of competing interest

The authors declare that they have no known competing financial interests or personal relationships that could have appeared to influence the work reported in this paper.
